# Association of proportion of the HDL-cholesterol subclasses HDL-2b and HDL-3 and macrovascular events among patients undergoing hemodialysis

**DOI:** 10.1038/s41598-021-81636-3

**Published:** 2021-01-21

**Authors:** Wen-Chin Lee, Jin-Bor Chen, Sin-Hua Moi, Cheng-Hong Yang

**Affiliations:** 1 Division of Nephrology, Department of Internal Medicine, Kaohsiung Chang Gung Memorial Hospital and Chang Gung University College of Medicine, DaPei Rd, Niao Song District, Kaohsiung, Taiwan; 2 Center of Cancer Program Development, E-Da Cancer Hospital, I-Shou University, Kaohsiung, Taiwan; 3 Department of Electronic Engineering, National Kaohsiung University of Science and Technology, Kaohsiung, Taiwan

**Keywords:** Medical research, Nephrology

## Abstract

Altered high-density lipoprotein cholesterol (HDL-C) subclass distribution in hemodialysis (HD) patients is well documented. Aim of this study is to investigate the relationship between HDL-C subclass distribution and macrovascular events in patients undergoing HD. A total of 164 prevalent HD patients and 71 healthy individuals in one hospital-facilitated clinic were enrolled from May 2019 to July 2019 and individual HD patients was follow-up for one year. Macrovascular events (cerebral stroke, coronary heart disease) were recorded in the study period. The HDL-2b, HDL-3 proportions and biochemical parameters were measured. Pearson correlation test and logistic regression analysis were used to examine correlation and odds ratio (OR). 144 HD patients completed one-year follow-up. Cohort with macrovascular events revealed significantly lower HDL-2b and higher HDL-3 subclass proportions compared to those without events. By multivariable adjustment, HDL-3 subclass proportion revealed significantly increase risk for these events (OR 1.17, 95% CI 1.02–1.41, *P* = 0.044). HDL-2b subclass was significantly higher and HDL-3 subclass was significantly lower in the HD cohort under the hs-CRP level of < 3 mg/L compared to higher hs-CRP level. In conclusion, HDL-2b and HDL-3 subclasses distributions were associated with macrovascular events in HD patients. Proinflammatory status influences the distribution of HDL-2b and HDL-3 subclasses in HD patients.

## Introduction

Patients with chronic kidney disease (CKD) are at high risk for cardiovascular diseases (CVD) and cerebral stroke (CS)^1,2^. Various traditional risk factors, such as dyslipidemia, diabetes mellitus and cardiac arrhythmia, possibly contribute to these events^1^. However, nontraditional factors, such as chronic inflammation, mineral abnormality and uremia, also increased the risk of developing these ecvents^3^.

It is well recognized that high-density lipoprotein cholesterol (HDL-C) is an independent predictor of CVD in the general population^4^. Dyslipidemia also has been considered as a major risk factor for CVD in the CKD population^5,6^. Screening for dyslipidemia in patients with CKD is advocated by clinical guidelines^4,7^. A well- known in CKD population is that HDL-C alters its composition and function. However, the association of HDL-C with CVD risk is still controversial in CKD population^8–11^.

HDL is composed of varying subclass particles, different in chemical characteristics and physicochemical properities^12,13^. By analytic measures, HDL can be divided into the following subclasses: HDL-2a, HDL-2b, HDL-3a, HDL-3b, HDL-3c, pre-beta_1_-HDL, and pre-beta_2_-HDL^13^. In prior studies, HDL subclasses contribute to different effects on atherosclerosis. An increased in the proportion of large particle, such as HDL-2b, exerts an antiatherogenic effect. In contrast, an increased in the proportion of smaller particle, such as HDL-3 and pre-beta_1_-HDL, is positively associated with CVD^12,14^. Due to the complex technique and instrument requirement, the measurement of HDL subclasses is not widely applied in the past years. Recently, a microfluidic chip-based technique is developed. This fast, easy-to-operate technique is successfully applied to measure the HDL subclasses in the epidemiological studies^15,16^.

We hypothesized that patients with hemodialysis (HD) have altered HDL subclasses distribution compared with healthy individuals. Consequently, this effect leads to an increase risk for macrovascular events in HD patients. Therefore, this study aimed to investigate the impact of HDL-2b and HDL-3 subclass distributions on the incidence of macrovascular events in a cohort of HD patients.

## Results

### Characteristics of participants

**Figure 1:**
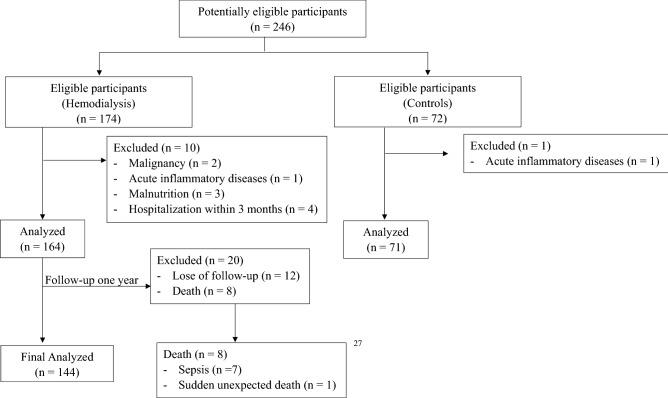
Participants flow diagram.

Participants were screening by inclusion and exclusion criteria. Finally, a total of 164 participants with HD and 71 healthy controls were included in the baseline analyses and 144 HD participants completed one-year follow-up (Fig. 1). The mean ages of participants with HD and healthy controls were 63.1 and 49.9 years, respectively. The proportions of male and female participants in the HD cohort and healthy controls were 48.8% and 51.2% and 31.0% and 69.0%, respectively.

In the comparison of lipid profiles, HD patients showed a significantly lower HDL-2b subclass proportion compared with healthy controls as well as lower total cholesterol, HDL-C and LDL-C levels. The HDL-3 subclass proportion was not significantly different between the HD patients and healthy controls (Table 1).

**Table 1:** Baseline characteristics in study cohort (N = 235).

Variables	HD (n = 164)	Control (n = 71)	*P*
Age, years	63.1 ± 12.3	49.9 ± 10.6	< 0.001
Sex			0.017
Female	84 (51.2%)	49 (69.0%)	
Male	80 (48.8%)	22 (31.0%)	
BMI, kg/m^2^	23.5 ± 12.5	23.3 ± 4.1	0.925
Dialysis vintage, years	10.48 ± 7.53	–	–
Diabetes	27 (16.5%)	N/A	–
Antihypertensive	66 (40.2%)	–	
Lipid-lowering drugs	24 (14.6%)	–	
Etiology of kidney failure			–
Primary kidney disease	61 (37.2%)	N/A	
Systemic disease	79 (48.2%)	N/A	
Unknown	24 (14.6%)	N/A	
Laboratory measurements			
Hemoglobin, g/dL	10.7 ± 1.2	12.6 ± 1.7	< 0.001
Total leukocytes, 10^9^/L	6.3 ± 2.4	6.1 ± 1.7	0.567
Albumin, g/dL	3.9 ± 0.3	4.2 ± 0.8	0.075
BUN, mg/dL	69 (32–151)	13 (7–46)	< 0.001
Cr, mg/dL	10.4 ± 2.3	0.7 ± 0.2	< 0.001
Total Cholesterol, mg/dL	152 (94–314)	189 (135–310)	< 0.001
Triglyceride, mg/dL	108 (30–994)	80.5 (18–237)	< 0.001
HDL-C, mg/dL	44.5 ± 15.4	59.8 ± 14.0	< 0.001
HDL-2b, %	23.6 ± 9.1	31.2 ± 6.7	< 0.001
HDL-3, %	31.7 ± 11	33.6 ± 7.5	0.137
LDL-C, mg/dL	88.1 ± 35.0	112.1 ± 30.8	< 0.001
Intact-PTH, pg/ml	268.2 (2.4–2819.9)	53.6 (20.9–90.2)	< 0.001
hs-CRP, mg/L	6.9 ± 13.0	2.7 ± 5.0	0.016

*BMI* body mass index, *HDL* high-density lipoprotein, *LDL* low-density lipoprotein, *C* cholesterol, *T* total, *PTH* parathyroid hormone, *BUN* blood urea nitrogen, *Cr* creatinine, *hs-CRP* high-sensitivity C-reactive protein.

Comparison of macrovascular events in HD participants based on baseline characteristics.

In one-year follow-up in HD participants, cohort with macrovascular events (CHD/CS) revealed significantly lower HDL-2b subclass proportion and higher HDL-3 subclass proportion compared to those without events (Table 2). Table 3 performed the logistic regression analysis results that evaluated the association between CHD/CS and baseline characteristics in HD patients. All demographic characteristics and laboratory measurements in Table 2 were included in both univariate and fully adjusted multivariate regression analyses. In univariate analysis results, both HDL-2b and HDL-3 showed no significant association with the risk of CHD/CS. However, the fully adjusted multivariate regression results indicated HDL-3 subclass proportion revealed significantly increase risk for CHD/CS events (odds ratio 1.17, *P* = 0.044) (Table 3). Hence, the inclusion of other demographics characteristics and laboratory measurements were potentially adjusted the association between HDL-3 and CHD/CS.

**Table 2:** Baseline characteristics in hemodialysis patients (N = 144).

Variables	CHD/CS (n = 27)	Non CHD/CS (n = 117)	*P*
Age, years	65.8 ± 13.6	62.3 ± 12.3	0.226
HD vintage, years	11.2 ± 8.1	10.7 ± 7.6	0.784
Sex			0.122
Female	9 (33.3%)	61 (52.1%)	
Male	18 (66.7%)	56 (47.9%)	
DM	8 (29.6%)	18 (15.4%)	0.145
Etiology			0.862
Primary kidney disease	8 (29.6%)	42 (35.9%)	
Systemic disease	15 (55.6%)	56 (47.9%)	
Others	4 (14.8%)	19 (16.2%)	
Laboratory measurements			
Hemoglobin, g/dL	11 ± 1.0	10.6 ± 1.3	0.139
Total leukocytes, 10^9^/L	6.7 ± 2.5	6.1 ± 2.4	0.290
Albumin, g/dL	3.9 ± 0.4	3.9 ± 0.3	0.930
BUN, mg/dL	73 (37–111)	68 (32–151)	0.339
Cr, mg/dL	10.6 ± 2.5	10.3 ± 2.3	0.661
Total cholesterol, mg/dL	143 (102–195)	151 (94–314)	0.069
Triglyceride, mg/dL	110 (53–290)	102 (30–300)	0.664
HDL-C, mg/dL	42.6 ± 13.4	45.8 ± 15.4	0.285
HDL-2b, %	20.0 ± 7.7	24.9 ± 9.1	0.006
HDL-3, %	34.9 ± 11.0	29.6 ± 9.8	0.026
LDL-C, mg/dL	78.6 ± 26.7	88.1 ± 34.1	0.122
Intact-PTH, pg/ml	199.7 (17.2–2819.9)	303.1 (2.4–2130)	0.865
hs-CRP, mg/L	8.7 ± 12.4	6.8 ± 13.9	0.469

*CHD* coronary heart disease, *CS* cerebral stroke.

**Table 3:** Logistic regression analysis for CHD/CS.

Variables	Univariate		Multivariate	
	OR (95% CI)	*P*	OR (95% CI)	*P*
Dependent variables: CHD/CS vs. Non CHD/CS				
Age, years	1.00 (0.95–1.06)	0.986	0.95 (0.85–1.04)	0.300
HD vintage, years	0.98 (0.89–1.07)	0.700	0.97 (0.83–1.10)	0.600
Sex				
Female	–		–	
Male	0.74 (0.18–2.92)	0.700	0.60 (0.06–5.27)	0.600
DM	4.11 (0.95–16.8)	0.047	1.07 (0.06–17.4)	0.959
Etiology				
Primary kidney disease/Others	–		–	
Systemic disease	3.88 (0.90–26.7)	0.100	3.99 (0.26–113)	0.300
Laboratory measurements				
Hemoglobin, g/dL	1.07 (0.62–1.83)	0.800	1.08 (0.45–2.47)	0.900
Total leukocytes, 10^9^/L	1.18 (0.94–1.44)	0.110	1.10 (0.68–1.58)	0.700
Albumin, g/dL	0.22 (0.03–1.41)	0.100	0.49 (0.01–28.4)	0.700
BUN, mg/dL	0.98 (0.94–1.02)	0.400	0.98 (0.92–1.05)	0.600
Cr, mg/dL	0.72 (0.51–0.98)	0.045	0.55 (0.19–1.10)	0.200
Total cholesterol, mg/dL	1.00 (0.99–1.01)	0.400	1.37 (0.70–3.08)	0.400
Triglyceride, mg/dL	1.00 (0.98–1.01)	0.800	0.19 (0.00–5.59)	0.400
HDL-C, mg/dL	0.94 (0.87–0.99)	0.060	4.63 (0.16–269)	0.400
HDL-2b, %	0.99 (0.91–1.07)	0.800	1.09 (0.93–1.33)	0.300
HDL-3, %	1.06 (0.99–1.14)	0.110	1.17 (1.02–1.41)	0.044
LDL-C, mg/dL	1.00 (0.98–1.02)	0.800	5.28 (0.18–322)	0.400
Intact-PTH, pg/ml	1.00 (1.00–1.00)	0.200	1.00 (0.99–1.00)	0.500
hs-CRP, mg/L	1.02 (0.98–1.05)	0.300	1.02 (0.93–1.08)	0.700

### Pearson correlation analysis on study parameters

The results from the Pearson correlation test showed that the HDL-2b subclass proportion was significantly negatively correlated with age, HDL-3 subclass proportion, triglyceride and total peripheral leukocyte count. On the other hand, it was significantly positively correlated with HDL-C, total cholesterol, and hemoglobin. The HDL-3 subclass proportion was significantly negatively correlated with the HDL-2b subclass proportion and HDL-C and positively correlated with triglyceride, hemoglobin, and total peripheral leukocyte count (Table 4).

**Table 4:** Pearson correlation analysis.

Variables	HDL-2b (%)	HDL-3
Age (years)	− 0.26***	0.01
HDL-2b (%)	1.00	− 0.54***
HDL-3 (%)	− 0.54***	1.00
HDL-C (mg/dL)	0.68***	− 0.52***
Cholesterol (mg/dL)	0.21**	− 0.03
Triglyceride (mg/dL)	− 0.44***	0.51***
LDL-C (mg/dL)	0.10	0.02
Intact-PTH (pg/ml)	< 0.001	− 0.03
Albumin (g/dL)	0.05	0.13
hs-CRP (mg/L)	− 0.11	< 0.001
Hemoglobin (g/dL)	0.16*	0.14*
Total leukocytes (10^9^/L)	− 0.16*	0.19**

*p < 0.05, **p < 0.01, ***p < 0.001.

Associations of the hs-CRP levels with the HDL-2b and HDL-3 subclass proportions in HD participants.

**Figure 2:**
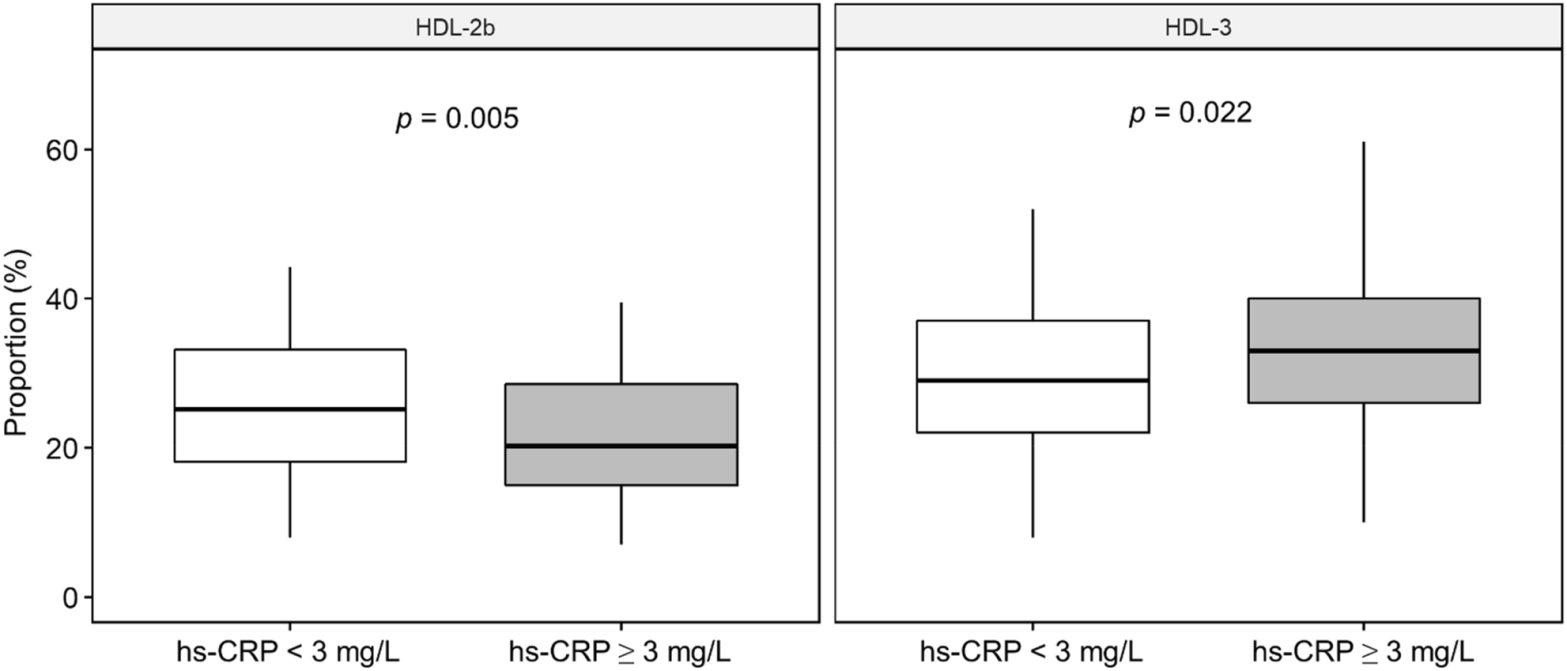
Proportion of the HDL-2b and HDL-3 subclasses in patients with hemodialysis stratified by an hs-CRP concentration 3 mg/L.

We stratified the entire HD cohort with the cutoff hs-CRP level of 3 mg/L (normal range < 3 mg/L in the laboratory) and examined the associations with the HDL-2b and HDL-3 subclass proportions in HD participants. The results showed that the HDL-2b subclass was significantly higher and HDL-3 subclass was significantly lower in the HD cohort under the hs-CRP level of < 3 mg/L compared to higher hs-CRP level (Fig. 2).

## Discussion

The key findings in our study are that HD participants presented lower HDL-2b and HDL-3 subclass proportions compared with healthy controls. Among HD participants, HDL-3 subclass proportion would increase risk for CHD/CS events. Moreover, HDL-2b and HDL-3 subclass distribution were influenced by proinflammatory status, stratified by hs-CRP levels. Our findings imply that potential utilization of the proportion of the HDL subclass analysis in clinical risk stratification for vascular events in HD patients. In this study, we prefer to use proportions of HDL subclass analysis instead of a simple subclass measurement to generalize across the whole spectrum of the HDL levels^17,18^. The main consideration is technique and cost-consuming in measurement of whole spectrum HDL proportion. Meanwhile, the microfluidic chip-based technique is easy to be applied in the clinical investigation and could forward to cause-effect relationship investigation.

CKD patients have different HDL particles that vary in function, composition, and metabolism^19^. A growing body of evidence has demonstrated that HDL in CKD decreased its function as anti-inflammatory, antioxidant, and endothelial protection^20,21^. The mechanisms are complex, but altered HDL composition in CKD is thought to be one of the significant points^5,22^. Acquired lecithin:cholesterol acyltransferase (LCAT) deficiency is a major reason for low blood HDL in CKD^23,24^. Moreover, pre-beta1-HDL metabolism is delayed in patients with CKD patients not on dialysis^25,26^. Result may progress residual renal function decline in CKD patients^25^. CKD patients initiating HD were reported greater abundance of HDL-associated proteins involving in atherosclerotic, lipid metabolism and inflammatory pathways^27^. The distribution of HDL subclasses in CKD is not yet uniform reported^19^. Two reports have described that HD patients have a lower HDL-3 cholesterol level and do not have a decrease in the HDL-2 cholesterol level compared with healthy individuals^28^. Another report observed contradictory results of increased HDL-3a and decreased HDL-2b subclass levels in patients with HD compared with healthy controls^29^. It is also reported that HD patients have decreased levels of both HDL-2 and HDL-3 cholesterol^30^ The discrepancies in the aforementioned studies may have resulted from either the different methods for HDL subclass measurement or statistical comparison in different dialysis populations and relatively small case number. In our study, we used the microfluidic chip-based technique more easily to examine the proportional distribution of the HDL subclasses in HD patients. This technique has been successfully used to measure the HDL subclasses in the Prospective Cardiovascular Munster study^15^. Our participants with HD showed decreased proportions of both HDL-2b and HDL-3 compared with healthy individuals. This result is in accordance with previous recognition regarding the alteration of HDL subclass distribution in patients with CKD^8,22^. Nevertheless, the cause-effect relationship of alteration in distributed proportions of the HDL-C subclasses and survival outcomes in patients with CKD still warrants further investigation in a population-based, longitudinal observational study.

CKD is in a proinflammatory status. In clinical practice, CRP is commonly used as a marker of proinflammatory status. In the prior report, hs-CRP level has been linked to malnutrition, inflammation, and atherosclerosis syndrome and is considered to be a risk factor for morbidity and mortality in patients with CKD^31^. In diabetic mouse model, CRP/oxLDL/β2GPI complex aggravated atherosclerosis by increasing lipid uptake^32^. In our study, we found that HDL-2b and HDL-3 subclass proportions were influenced by the hs-CRP levels in HD participants. Lower hs-CRP levels were significantly associated with higher HDL-2b and lower HDL-3 subclass proportions. Accordingly, proinflammatory status could suppress the HDL-2b and increase HDL-3 subclass proportions in patients with HD. However, the relationship between the severity of proinflammatory status and HDL subclass distribution still needs to be determined in a future investigation.

Several factors have been implicated in vascular injury in CKD. The factors involves age, obesity, anemia, diabetes, calcium/phosphorus imbalance, proinflammatory status and dyslipidemia^33^. In vessels of CKD population, there commonly exists atherosclerosis and vascular calcification. Although distinguish risk factors in individual pattern, these vascular phenomenon confer an untoward outcomes in CKD. Consequently, CKD and dialysis patients present a high risk for macrovascular events including CHD and CS^2,34,35^ HDL plays a major role to against the development and progression of atherosclerosis. Alteration of HDL composition and function have been documented in CKD^36^. Low lecithin:cholesterol acyltransferase (LCAT) and paraoxonase activity in CKD diminish the conversion of HDL 3 to HDL2^36,37^. Cholesterol ester is transferred to low density lipoprotein (LDL) and very low density lipoprotein (VLDL) when HDL-2b is absent and results in increasing atherogenic particles, cholesterol ester. In addition, uremia-association inflammation causes oxidation of HDL and HDL subfraction abnormalities^36^. Consequently, CKD patients are predisposed to atherosclerosis. It is well recognized that a decrease content of HDL-2b are positively associated with the risk of CHD^14,38^. In present study, we found that HD participants with CHD/CS events had significantly lower HDL-2b subclass proportion and higher HDL-3 subclass proportion compared to those without CHS/CS event. This result is in line with reports in general population^12,14^ Moreover, we found that circulating triglyceride level was negatively correlated to HDL-2b subclass proportion and positively correlated to HDL-3 subclass proportion. We hypothesize that decreasing cholesterol ester donation from HDL-2 to VLDL remnants in exchange for triglyceride^36^. Taken together, abnormalities in HDL subclass distribution contribute to atherogenic burden in HD patients and increase a risk for CHD/CS events.

Our study has several limitations. First, this was a small-sized, single-center study on an Asian HD population. The results may not be extrapolated to other ethnic HD populations. Second, our study only measured the HDL subclass proportion in HD patients in one time point. The longitudinal effect of HD on HDL subclass distribution cannot be obtained in our study. Third, we did not measure whole HDL subclass proportion, therefore, impact of individual subclass cannot be obtained in this study. Finally, a subset of our cohort received lipid-lowering drugs during the study period. There may be a potential influence of the drug therapy on the HDL-C level or HDL particle distribution. Despite the aforementioned limitations, the strength of our study is that our results were comparable to those of previous studies^19,28–30^ that have examined the proportions of HDL subclasses, HDL-2b and HDL-3, in HD patients. Thus, our study provides cause-specific data to fill in knowledge gap on the reasons of HDL subclass distribution in these patients. The clinical utility of HDL subclass distribution analysis would be further facilitated by spanning a full-range HDL-C population and determine the subclass distribution on adverse macrovascular events.

In conclusion, HD patients have lower HDL-2b and HDL-3 subclass proportions compared with healthy individuals. The distribution of the HDL-2b and HDL-3 subclasses is influenced by proinflammatory status. Finally, these distribution contribute to the incidence of macrovascular events in HD patients.

## Methods

### Study design and participants

Adult patients (> 18 years) who underwent maintenance HD thrice weekly for at least 3 months in the outpatient clinic in Kaohsiung Chang Gung Memorial Hospital in Taiwan from May 2019 to July 2019 were enrolled in this study. The exclusion criteria were as follows: ongoing treatment for malignancy, acute inflammatory diseases, hospitalization within 3 months, malnutrition defined by serum albumin level < 3.5 g/dL, and pregnancy. Healthy controls were recruited voluntarily in the outpatient clinic by posted protocol notification. All HD participants were follow-up for one year. Informative patient data, including demographic profiles, laboratory parameters and macrovascular events [coronary heart disease (CHD), cerebral stroke (CS)] in the study period were collected.

The protocol for the study was approved by the Committee on Human Research at Kaohsiung Chang Gung Memorial Hospital (IRB document: 201801486B0) in Taiwan and conducted in accordance with the principles of the Declaration of Helsinki. All participants signed informed consent to approve study initiation.

All data supporting the study is presented in the manuscript or available upon request from the corresponding author of this manuscript, Jin-Bor Chen.

### Analytic parameters

All blood samples from HD participants in the fasting status and in mid-week (Wednesday and Thursday) were obtained. Blood samples for biochemistry measurement were obtained using commercial kits and an autoanalyzer (Hitachi 7600-210, Hitachi Ltd., Tokyo, Japan). Albumin levels were measured using the bromocresol green method. Intact parathyroid hormone level was measured using a chemiluminescence immunoassay (Siemens Healthcare Diagnostics Inc., USA)^39^. The high-sensitivity C-reactive protein (hs-CRP) level was assayed using the immunoturbidimetric method (Spectra East Laboratories, Rockleigh, NJ, USA)^40^. The plasma total cholesterol, triglyceride, and HDL-C levels were determined enzymatically on the Eroset Hitachi 7600-210 analyzer. The low-density lipoprotein cholesterol (LDL-C) levels were calculated using to the Friedewald formula^41^, which provides reliable values up to a triglyceride level of 4.0 mmol/L.

HDL-C subclass profiles were measured by electrophoresis of a microfluidic chip system. Briefly, serum samples, calibrator, and QC materials were diluted 1:50 in sample buffer in the presence of a mixture of lipophilic fluorescent dyes and allowed to incubate for 5–15 min prior to loading on to chips. Separation was carried out in a microfluidic device (MICEP-30, Ardent BioMed). The entire procedure was performed in less than 1 h. The HDL-2b and HDL-3 subclasses were automatically calculated in line by a proprietary algorithm (Ardent BioMed LLC, Mt. View, California, USA)^15^.

### Statistical analysis

The baseline demographic characteristics and laboratory measurements in HD patients and healthy controls are presented as frequency (percentage) and mean (standard deviation). The distribution difference was estimated using the independent two-sample t-test or chi-square test. Logistic regression analysis was performed to evaluate the association between CHD/CS and baseline characteristics in HD patients. The demographic characteristics including age, HD vintage, sex, DM, etiology and laboratory measurements were included in univariate logistic regression model. All included variables were retained in multivariate logistic regression model in order to further explore association between CHD/CS and HDL-2b, HDL-3 after adjusted common covariates in HD patients. The correlation between the HDL-2b and HDL-3 proportions and associated variables was estimated using the Pearson correlation test. All *P* values were two-sided, and *P* < 0.05 was considered statistically significant. All statistical analyses were performed using the R 3.6.3 software (R Core Team, 2019)^42^.
